# AD diagnosis model based on fusion of heterogeneous brain imaging and genomic data

**DOI:** 10.3389/fnins.2026.1719390

**Published:** 2026-03-06

**Authors:** Zhihao Zhang, Ruixia Zhang, Wenzhong Yang, Ke lv, Miao Wu, Lianghui Xu

**Affiliations:** 1 School of Computer Science and Technology, Xinjiang University, Urumqi, China; 2 Institute of Medical Engineering Interdisciplinary Research, Xinjiang Medical University, Urumqi, China

**Keywords:** Alzheimer's disease diagnosis, bioinformatics, early screening diagnosis, machine learning, multimodal fusions

## Abstract

Alzheimer's disease (AD) is a common neurodegenerative disorder in the elderly population, and early screening can effectively delay the progression of the disease. Mild cognitive impairment (MCI) occurs prior to the onset of AD; however, the accuracy of existing MCI-to-AD prediction methods remains relatively low. Additionally, small sample sizes and high feature dimensions often lead to model overfitting, highlighting the need for effective early screening approaches. To address the aforementioned issues, this study integrated non-paired multi-modal features—including clinical indicators from the ADNI database, blood biomarkers, brain region volume features extracted from MRI, and genetic biomarkers from the GEO database—and proposed a gender-corrected random matching strategy. The Random Forest algorithm was adopted to evaluate this strategy, analyze feature importance, and compare the performance of 9 machine learning algorithms based on the top 40 ranked features. The predictive performance of multi-modal data was superior to that of single-modal data, and the proposed strategy achieved favorable results in early AD screening. 16 specific genetic features (e.g., IFI27, EDF1, RAP2A, KIF5C, SERPINA3, FBXW7, IFITM1, ISG15, PSMB3, APOE4, KCNB1, PSPH, HMGN2, S100A13, IFIT3, and CALM1) and 6 brain region volume features ranked high in terms of importance. When validated using paired datasets from ADNI across the 9 algorithms, ensemble learning models demonstrated significantly stronger fitting capabilities. The non-paired multi-modal fusion approach not only expands the sample size but also enhances the generalization ability and robustness of the model. This provides a theoretical basis for the application of this strategy in the field of small-sample medical research.

## 1 Introduction

Alzheimer's Disease (AD) is an irreversible neurodegenerative disorder caused by the accumulation of extracellular beta-amyloid (Aβ) and intracellular neurofibrillary tangles, primarily affecting the elderly and lead to severe impairments in memory, thinking, language, and daily living skills (Hane et al., 2017). According to 2019 statistics from the World Health Organization, AD and other forms of dementia are among the top ten causes of death worldwide. Patients require long-term treatment and care management, significantly impacting their family lives and incurring substantial societal costs.

In the asymptomatic phase of AD, some patients may experience spontaneous reversal without special treatment. However, once in the dementia phase, even with active treatment, the disease cannot be reversed, and only its progression can be slowed. Some patients may reverse with reasonable and effective treatment during the mild cognitive impairment (MCI) phase. Therefore, treating AD in its preclinical phase is the best opportunity to slow disease progression (Rafii and Aisen, 2023), making early screening and diagnosis crucial.

Imaging techniques are commonly used for diagnosing Alzheimer's Disease, with studies on brain metabolism using MRI and positron emission tomography revealing characteristic changes in AD patients (Prasath and Sumathi, 2023). Haleem et al. (2019) and Dimitriadis et al. (2018) manually extracted features from multiple brain images using neuroimaging processing tools (such as FreeSurfer, MIPAV, FSL, and SPM), and then used machine learning classifiers to achieve AD classification (Kruthika and Rajeswari, 2019; Uysal and Ozturk, 2020). Deep learning, especially Convolutional Neural Networks (CNNs), can effectively extract underlying patterns from data and have achieved significant success in AD diagnosis. Typical CNN architectures such as LeNet (Sarraf and Tofighi, 2016), AlexNet (Lee et al., 2019; Kazemi and Houghten, 2018), GoogleNet (Ding et al., 2019), VGGNet (Mehmood et al., 2020), ResNet (Yee et al., 2019), and DenseNet (Wang et al., 2019) have been widely used in early AD diagnosis. Although deep learning methods have high predictive accuracy in disease diagnosis, they have limitations in interpretability. The decision-making process remains challenging in clinical practice, and the accuracy in identifying patients with mild cognitive impairment is not high.

With the continuous advancement of high-throughput testing technology, a large amount of multi-omics data has been accumulated. Researchers are dedicated to identifying and diagnosing key biomarkers for AD. Methods such as genome-wide association studies (Liu et al., 2022), differential gene analysis (Madar et al., 2021), gene co-expression network analysis (Zhang and Kiryu, 2023), and machine learning (Liu et al., 2023) are used to screen for key genes. The expression levels of these key genes are then used as input features to construct AD diagnostic models.

**Figure 1:**
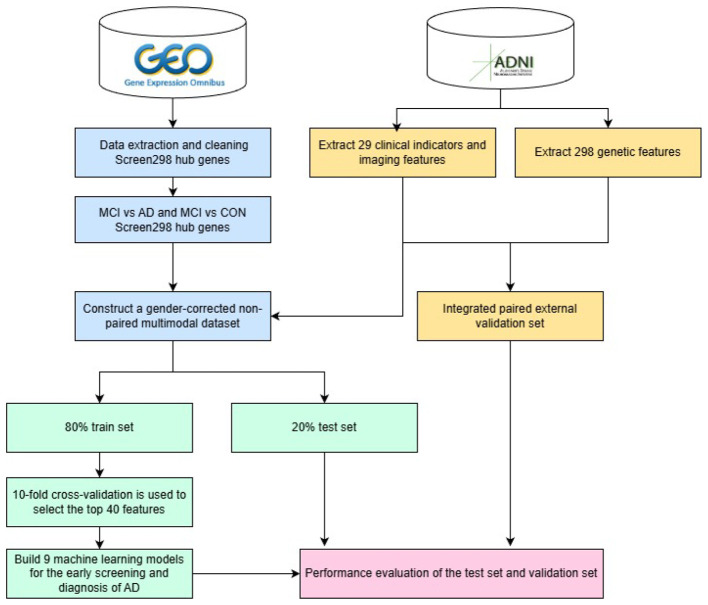
Overall workflow of the study.

Some researchers have constructed multimodal AD diagnostic models by integrating different modalities of data. For example, Dai et al. (2019) combined the results of an improved LeNet-5 model based on sMRI, PET, and multimodal fusion images with the Bayesian method classification results based on the Clinical Dementia Rating Scale, demonstrating that multimodal auxiliary diagnostic methods can achieve good diagnostic results (Zhang et al., 2019). Wu et al. (2023) integrated demographic data, neuropsychological tests, and MRI-related biomarker features to apply machine learning models to predict the progression from MCI to AD. Gaubert et al. (2021) combined non-invasive features (EEG, APOE4 genotype, demographic data, neuropsychological, and MRI data) for AD prediction tasks, finding that multimodal biomarkers are helpful for early AD diagnosis. Dobromyslin and Megherbi (2022) proposed a method of combining gene expression data from multiple platforms, constructing an AD prediction model based on differentially expressed genes and imaging biomarkers, which showed higher accuracy than models using only imaging biomarkers. Yang et al. (2023) utilized metabolic biomarkers and demographic variables to construct logistic regression and random forest-based AD diagnostic models, providing scientific references for early screening and diagnosis of AD. Pan et al. (2023) found that an MCI diagnostic model combining plasma biomarkers, cognitive test scores, and demographic characteristics outperformed models using only plasma biomarkers. Wang et al. (2023) found that a composite information biomarker panel, combining biomarkers and clinical information, significantly improved the staging performance of AD progression. Zhang et al. (2025) evaluated the PID metric method in multimodal data fusion, proposing improvements to enhance its accuracy. Huang et al. (2025a) proposed the MAFDSRP model, which combines neuroimaging and genetic data to identify biomarkers associated with Alzheimer's disease. Huang et al. (2025b) introduced the MFIFN framework, which improves Alzheimer's disease classification accuracy by integrating fMRI and sMRI data. Wu et al. (2025) proposed the KAGAN model, combining graph attention mechanisms with clinical information to optimize AD classification performance. Many research results have shown that diagnostic models based on multimodal data fusion have higher accuracy and better robustness compared to single-modal diagnostic models. Therefore, the development of early AD screening models using multimodal data fusion is an important research direction. However, multimodal data present challenges in terms of collection and processing difficulties, high costs, and issues with data consistency and matching. In response to the dual impact of the aforementioned data matching challenges and pathological modification factors, this paper proposes a gender-corrected early screening and diagnosis scheme for AD that adapts to unmatched multimodal omics data from the perspective of pathological mechanisms, and tests the performance of this method on paired data. The specific process is shown in Figure 1. The main contributions of this study are as follows:

(1) Addressing the Early Screening Diagnosis Challenges for MCI Patients: Using the GSE84422 gene expression data as the research subject, two control experiments (MCI-CON and MCI-AD) were constructed. Differential gene analysis and gene co-expression network analysis methods were used to screen hub genes across data from 19 brain regions, and the functional analysis of MCI-related gene features was conducted.
(2) Proposing a Gender-Corrected Multimodal Data Random Matching Strategy: To address the scarcity of matched multimodal data and the difficulty of sample collection, a gender-corrected random matching strategy for multimodal data was proposed. This strategy integrates gene features mined from GEO data with demographic characteristics, clinical indicator features, mental state examination features, and neuroimaging features from ADNI data.
(3) Building an AD Early Screening Diagnostic Model: The integrated features were used as input to construct an AD early screening diagnostic model based on the Random Forest algorithm. Feature contribution analysis revealed that the volume features of the Entorhinal, Hippocampus, and Fusiform brain regions, along with 16 gene features (IFI27, EDF1, RAP2A, KIF5C, SERPINA3, FBXW7, IFITM1, ISG15, PSMB3, APOE4, KCNB1, PSPH, HMGN2, S100A13, IFIT3, and CALM1), were among the top contributors.
(4) Comparing Machine Learning Algorithms: Using the top 40 contributing features as new inputs, we compared the predictive performance of nine machine learning algorithms in the AD early screening diagnostic task.

## 2 Data and methods

### 2.1 GEO dataset

This study downloaded the GSE84422 (Wang et al., 2016) dataset from the Gene Expression Omnibus (GEO) at the National Center for Biotechnology Information (NCBI).^1^ This dataset includes 1053 samples from 19 brain regions of 125 patients with varying degrees of dementia. The diagnostic labels include “Normal,” “Possible AD,” “Probable AD,” and “Definite AD.”

### 2.2 Gene expression data preprocessing

The GSE84422 dataset was downloaded using the GEOquery package. Initially, the data was grouped based on 19 brain region categories, followed by preprocessing steps. The preprocessing tasks included: gene annotation based on platform probe information, retaining the first gene name when a probe mapped to multiple gene names, averaging expression values for duplicated gene names, removing records containing missing values, retaining the top 40% of gene expression records, and normalizing using the limma package.

Additionally, to maintain consistency with the ADNI dataset's category labels, we merged the diagnostic labels as follows: “Normal” was labeled as CON, “Probable AD” and “Possible AD” were labeled as MCI, and “Definite AD” was labeled as AD. The dataset included 362 AD samples, 439 MCI samples, and 252 CON samples. After grouping by different brain regions and cleaning the data, the resulting gene data is shown in Table 1. It is important to note that this study focuses on improving the diagnostic accuracy for the MCI category by constructing two comparison groups: MCI-CON and MCI-AD.

**Table 1:** Statistics of cleaned data samples.

**Sample type**	**AD**	**MCI**	**CON**	**Sample**
Amygdala	17	19	15	51
Anterior cingulate	20	23	16	59
Caudate nucleus	18	23	11	52
Dorsolateral prefrontal cortex	17	24	16	57
Frontal pole	24	24	15	63
Hippocampus	18	26	11	55
Inferior frontal gyrus	19	23	11	53
Inferior temporal gyrus	19	25	14	58
Middle temporal gyrus	20	14	24	58
Nucleus accumbens	17	21	13	51
Occipital visual cortex	14	26	13	53
Parahippocampal gyrus	23	22	15	60
Posterior cingulate cortex	23	23	12	58
Precentral gyrus	19	25	5	49
Prefrontal cortex	21	24	11	56
Putamen	20	23	9	52
Superior parietal lobule	13	24	13	50
Superior temporal gyrus	22	24	14	60
Temporal pole	18	26	14	58

### 2.3 ADNI database

The Alzheimer's Disease Neuroimaging Initiative (ADNI) database^2^ was initiated in 2004 by the National Institute on Aging, the National Institute of Biomedical Imaging and Bioengineering, the Food and Drug Administration, private pharmaceutical companies, and non-profit organizations. This $60 million, 5-year public-private partnership aims to test whether serial MRI, PET, other biological markers, and clinical and neuropsychological assessments can be combined to measure the progression of MCI and early AD.

This study includes data from 1530 individuals from the ADNI 1, ADNI 2, and ADNI GO cohorts. The ADNI dataset provides a comprehensive set of multimodal data, including demographic information, clinical indicators, mental state examination scores, and neuroimaging features, which are critical for integrating with gene expression data for a robust AD early screening diagnostic model.

### 2.4 ADNI data pre-processing

We utilized the longitudinal data stream of FreeSurfer 6.0 to process images through a fully automated workflow. Using the Destrieux (2009) brain atlas, we identified 74 anatomical regions of interest (ROIs) in the gray matter of each hemisphere. Notably, we standardized the regional volumes by intracranial volume (ICV) to compensate for individual differences in brain morphology and overall head size.

To visualize disease states, tissue density maps were calculated using the Destrieux atlas. Each image was first registered to a single brain template and segmented into gray and white matter tissues. The Destrieux maps provided local and separate encoding for each tissue type, and volume changes were observed during the registration process. Finally, gray matter volume features were extracted for six brain regions: Ventricles, Whole Brain, Hippocampus, Entorhinal, Fusiform, and Middle Temporal (MidTemp).

The retained sample data included clinical indicators (cognitive tests) and T1-weighted volumetric MRI scans. Samples with any missing data patterns were excluded. Participants were included in the study if they had at least two T1-weighted volumetric MRI scans. To ensure the selection of informative clinical variables and dilute the correlation between variables, only clinical variables with a missing rate of less than 30% were included. The Multivariate Imputation by Chained Equations (MICE) method was used to fill in data with a missing rate of less than 30%, utilizing the R package MICE (version 3.14).

After performing deletion of missing values and imputation, the preprocessed dataset (Dataset2) contained 2,186 records with the following patient phenotypes: 635 CON records, 1,043 MCI records, and 508 AD records. The dataset included 27 features, specifically: age, gender, APOE4 gene carrier status, three cerebrospinal fluid biomarkers (ABETA, TAU, and PTAU), 15 features from mental state examination and clinical dementia rating scales, and gray matter volume features from six brain regions extracted using FreeSurfer.

We downloaded gene expression data from the ADNI database, which includes 745 samples with a total of 49,387 original gene features. The gene expression data was inner joined with Dataset2 based on patient identifiers to form the external validation set (Dataset5). This resulting dataset contains 148 paired samples with the following patient phenotypes: 22 CON records, 111 MCI records, and 15 AD records. Dataset5 consists of 325 features.

### 2.5 Differential expression analysis

Differential expression analysis is one of the simplest methods for identifying potential biomarkers by searching for differential gene data between different phenotypes. We used the limma package to perform differential expression analysis for each of the 19 brain regions separately.

Using the “lmFit” function provided by Limma, linear models were fitted to the normalized data. After model fitting, the “eBayes” function in limma was used to calculate empirical Bayes moderated t-statistics and *p*-values for each gene. Thresholds for filtering differentially expressed genes were set as follows: |logFC|>1.5 and ρ < 0.05.

Finally, comparison experiments were set up between the MCI and CON groups and between the MCI and AD groups. Differentially expressed genes meeting the threshold criteria were compiled for each of the 19 brain regions.

### 2.6 Weighted gene co-expression network analysis

Weighted gene co-expression network analysis is a typical method for studying gene co-expression, which explores the relationships between genes with similar expression patterns and external clinical information by constructing scale-free co-expression networks. In a gene co-expression network, nodes represent genes, and edges represent the degree of their co-expression. We used the “WGCNA” package to construct the co-expression network. The specific steps of the experiment included:

(1) Hierarchical Clustering and Outlier Detection: Construct a hierarchical clustering tree to observe and remove outlier samples.
Adjacency Matrix Construction: Select a soft-thresholding power of 8 to convert the similarity matrix into an adjacency matrix. The co-expression similarity *S*_*ij*_ between genes i and j is defined in the adjacency matrix. The adjacency is calculated based on the Pearson correlation coefficient |*cor*(*x*_*i*_, *x*_*j*_)| between the expression profiles of genes i and j, where $a_{ij}=S_{ij}^{\beta }$ and β is a soft-thresholding power greater than or equal to 1, determined by the scale-free topology criterion.
(2) Topological Overlap Matrix (TOM): Construct the TOM to measure the average network connectivity of each gene.
(3) Dynamic Tree Cutting: Set the parameters deepSplit to 2 and minModuleSize to 20, and use the dynamic tree cut method to divide genes with similar expression profiles into different modules, setting the cutHeight value to 0.9.
(4) Module Eigengene Calculation: Construct a dendrogram through hierarchical clustering and calculate the module eigengenes (MEs) and their correlation with traits. For the 19 brain regions in the two control experiments, retain the gene modules with the highest correlation in the gene co-expression network.

Through these steps, the WGCNA analysis identifies gene modules exhibiting the strongest associations with the phenotype of interest while retaining genes expressed in each brain region.

### 2.7 GO enrichment analysis

GO analysis is widely used to describe the biological attributes of genes and gene products associated with specific biological processes (BPs), molecular functions (MFs), and cellular components (CCs). BPs involve a wide range of processes. We used the R package clusterProfiler to perform GO pathway analysis. The GO enrichment results were visualized using bubble charts plotted with the Chiplot function. A *p*-value of less than 0.05 was considered significant.

### 2.8 Protein-protein interaction network analysis

Protein-protein interaction (PPI) networks are composed of proteins and their interactions. We used the STRING database to construct a PPI network related to the MRDEGs (most relevant differentially expressed genes) with a minimum required interaction score of medium confidence (0.400).

### 2.9 Multimodal data fusion

Based on the core idea of the statistical consistent matching score method, this study implements random matching for unpaired samples of different modalities. It is well established that gender is a key modifier regulating the occurrence and progression of AD (Castro-Aldrete et al., 2025; Lopez-Lee et al., 2024). To reduce noise introduced by population heterogeneity, a gender stratification design was added during the random matching process. Using the hub gene feature dataset (Dataset1) screened from GEO data and the clinical indicator and imaging feature dataset (Dataset2) from ADNI data as the basis, an unpaired multimodal dataset (Dataset3) matched only by patient category labels and a gender-corrected multimodal dataset (Dataset4) matched by both category labels and gender were constructed respectively. Meanwhile, a paired multimodal dataset containing the aforementioned corresponding features was extracted from the ADNI database as the external validation set (Dataset5) for the model.

### 2.10 Model construction

This study verified the effectiveness of the multimodal data fusion strategy and screened the optimal model through multi-stage modeling. Firstly, Random Forest classification models were constructed for each of the 4 datasets. All datasets were divided into training sets and test sets at a ratio of 8:2, and the models adopted default hyperparameters. To address the class imbalance problem, the SMOTE algorithm was only applied to the training sets for oversampling. Average accuracy, balanced accuracy, and macro F1-score were calculated through ten-fold cross-validation to compare the modeling performance of different datasets.

Subsequently, optimization was performed exclusively for Dataset4: the training set was used for feature selection, and the independent test set was used to verify feature effectiveness. Feature selection was completed within the 10-fold cross-validation framework. For each fold, after applying SMOTE oversampling only to the sub-training set, feature importance was calculated using Random Forest. Based on the top 40 stable features obtained from the 10 importance ranking results, they are retained.

Finally, with stable features as inputs, a total of 9 machine learning models were constructed, including Logistic Regression, K-Nearest Neighbors (KNN), Decision Tree, Random Forest, AdaBoost, Gradient Boosting, Support Vector Machine (SVM), Extreme Gradient Boosting (XGBoost), and Light Gradient Boosting Machine (LightGBM). All models were tuned based on the training set of Dataset4, and their performance was initially evaluated on the independent test set. Further verification of generalization ability was conducted using the external validation set (Dataset5). Evaluation metrics included accuracy, Weighted precision, recall, and F1-score, ultimately screening out the optimal AD diagnostic model.

## 3 Result

### 3.1 Results of differential gene

**Figure 2:**
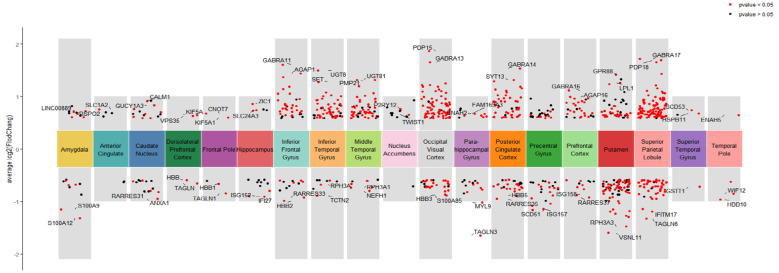
Volcano plots of differentially expressed genes in different brain regions for the MCI-CON group.

**Figure 3:**
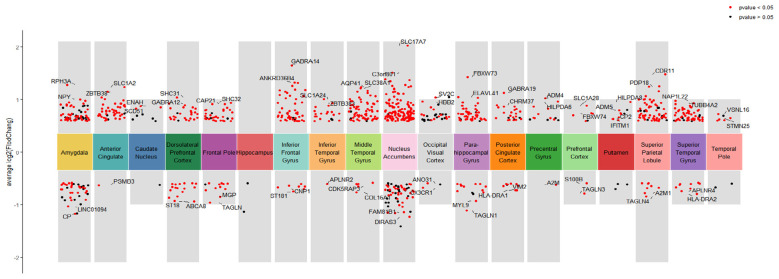
Volcano plots of differentially expressed genes in different brain regions for the MCI-AD group.

The volcano plots for the differential analysis of the MCI-CON group and the MCI-AD group across 19 brain regions are shown in Figures 2, 3. In the MCI-CON group, a total of 342 differentially expressed genes were identified, with the Superior Parietal Lobule brain region having the highest number of differentially expressed genes, totaling 153. In the MCI-AD group, 426 differentially expressed genes were found, with the Nucleus Accumbens brain region exhibiting the highest number, amounting to 169.

It is noteworthy that significant variations in the number of differentially expressed genes were observed between the two groups in brain regions such as the Nucleus Accumbens, Occipital Visual Cortex, and Putamen.

### 3.2 Results of WGCNA

Weighted gene co-expression network analysis (WGCNA) was conducted on the MCI-CON group and MCI-AD group across 19 brain regions. Based on the correlation coefficients of different gene modules, the gene module with the highest correlation coefficient was extracted for each group. In the MCI-CON group, the gene modules across the 19 brain regions contained a total of 2,077 genes, while in the MCI-AD group, the gene modules contained a total of 2,446 genes. Given the large total number of genes across the 19 brain regions, we considered the potential associations between brain regions and identified genes as highly correlated if they were co-expressed in at least two brain regions. Ultimately, there were 491 highly correlated genes in the MCI-CON group and 895 highly correlated genes in the MCI-AD group.

### 3.3 Analysis results of PPI networks

The union of the 342 differentially expressed genes from the MCI-CON group and the 491 highly correlated genes resulted in a total of 935 genes. Similarly, the union of the 426 differentially expressed genes from the MCI-AD group and the 895 highly correlated genes resulted in a total of 982 genes.

Protein-protein interaction (PPI) network analysis was performed on the genes selected from each group. This analysis identified 174 hub genes in the MCI-CON group and 258 hub genes in the MCI-AD group.

### 3.4 Enrichment analysis results

The union of the 174 hub genes from the MCI-CON group and the 258 hub genes from the MCI-AD group resulted in a total of 369 genes. GO enrichment analysis was performed on these combined genes, revealing that the differentially expressed genes are enriched in key pathways such as synaptic signaling, neurotrophic factors, and associative learning.

### 3.5 Multimodal data fusion

Through the analysis of the GEO dataset, a total of 369 hub genes were identified, with 301 of these genes being covered in the gene expression data across the 19 brain regions. To ensure complete coverage in the ADNI external validation dataset, the names of these 301 hub genes were intersected with the gene names in the ADNI database. This intersection resulted in the identification of 298 gene features in the external validation set, with the expression data for three genes (“ERCC5,” “ICE1,” “SEC22B”) not being matched. Consequently, the GEO gene expression dataset (Dataset1) ultimately includes 298 gene features.

The GEO gene expression dataset was randomly merged with the ADNI clinical and imaging data based on sample category labels to complete feature fusion, resulting in a randomly matched multimodal dataset (Dataset3). Given the significant association between AD onset and gender, the samples were further randomly merged based on category labels and gender information, creating a gender-adjusted randomly matched multimodal dataset (Dataset4). It is noteworthy that the number of female samples in the MCI category of the GEO dataset exceeded that in the ADNI clinical dataset, resulting in a final matched sample count of 1,019, which led to a reduction in the dataset size.

Additionally, we considered early-onset AD, defined as AD in patients under the age of 65, attempting to construct an age-adjusted randomly matched multimodal dataset. However, due to the scarcity of early-onset AD cases, the final sample size was less than 100, leading us to abandon the modeling exploration for this dataset. Table 2 presents detailed information about the constructed dataset.

**Table 2:** Descriptions of datasets used for modeling.

**Dataset ID**	**Data source**	**Sample size**	**Dataset details**
Dataset1	GEO	1,053	298 gene features retained after retrieving and matching 369 candidate genes with ADNI database
Dataset2	ADNI	2,186	21 clinical indicators and 6 brain region volume features extracted from imaging data (27 features: 21 clinical features + 6 brain region volume features)
Dataset3	GEO + ADNI	1,053	Multimodal dataset constructed by matching non-paired samples of Dataset1 and Dataset2 based on diagnostic labels (298 gene features + 21 clinical features + 6 brain region volume features)
Dataset4	GEO + ADNI	1,018	Multimodal dataset constructed by matching non-paired samples of Dataset1 and Dataset2 based on diagnostic labels and gender (298 gene features + 21 clinical features + 6 brain region volume features)
Dataset5	ADNI	148	Paired samples extracted from ADNI database, used as the external validation set (298 gene features + 21 clinical features + 6 brain region volume features)

### 3.6 Results analysis of the AD early screening diagnostic model based on random forest

An early AD screening model was developed using Random Forest methodology. Mean cross-validation results across datasets are presented in Table 3.

**Table 3:** Average prediction results of 10-fold cross-validation for four datasets.

**Model**	**Dataset**	**Accuracy**	***p***	**r**	**f1**	**Support**
Model1	Dataset1	0.7174	0.7260	0.7174	0.7200	270
Model2	Dataset2	0.9248	0.9262	0.9248	0.9250	626
Model3	Dataset3	0.9378	0.9390	0.9178	0.9372	270
Model4	Dataset4	0.9385	0.9386	0.9385	0.9385	270

As demonstrated in Table 3, unimodal gene expression data (Dataset1) yielded suboptimal diagnostic performance (Accuracy = 71.7%). Integration of clinical indicators, neuropsychological assessments, and neuroimaging biomarkers from ADNI (Dataset2) achieved superior discrimination (Accuracy = 92.5%). Stochastic pairing fusion (Dataset3) further enhanced predictive capability (Accuracy = 93.8%), while gender-adjusted multimodal integration (Dataset4) maintained robust accuracy (93.9%) despite moderate sample size reduction.

According to the prediction results in Table 3, it can be seen that the prediction performance is very low when using the single-modality gene expression data (Dataset1) for AD diagnosis. The clinical indicators, examination scale features, and extracted imaging features in the ADNI database have high quality, and using Dataset2 for modeling achieves a prediction accuracy of up to 92%. When Dataset1 and Dataset2 are merged, the prediction performance improves to some extent. The prediction results on the gender-adjusted multimodal dataset (Dataset4) show that although the sample size is slightly reduced, the prediction accuracy remains stable at around 93%. The comparison results of different datasets have verified the feasibility of the randomly matched multimodal dataset constructed in this study.

### 3.7 Feature selection and feature importance analysis

**Figure 4:**
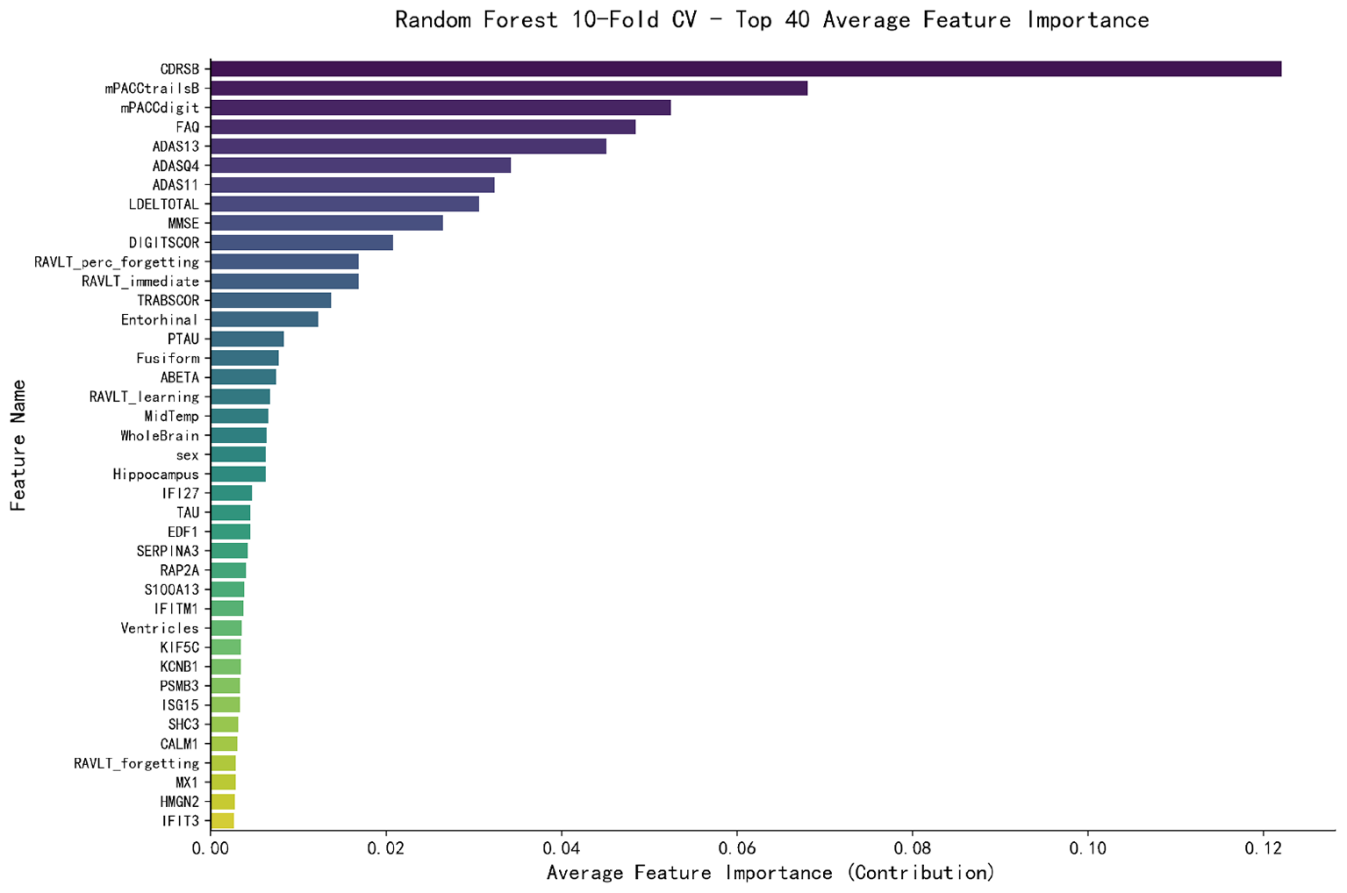
Bar chart of the top 40 feature contributions in Model4.

In the training set of Dataset4, feature selection was carried out, and the top 40 features with the highest average importance as generated by 10-fold cross-validation were obtained. We analyzed the feature importance of Model4, with Figure 4 displaying the bar chart of the top 40 contributing features. It is evident from the figure that the neuropsychological assessment scales from the ADNI database have the highest contributions, occupying all the top 10 positions. Their combined contribution reaches 48.14%, exerting a decisive impact on the model's predictive performance. Among these scales, CDRSB ranks first with an average importance of 0.1220, nearly twice that of mPACCtrailsB (0.0680) in the second place. The brain region volume features derived from imaging data, such as Entorhinal, Fusiform, and Hippocampus, are ranked 14th, 16th, and 22nd, respectively, with MidTemp and WholeBrain also listed in the top 20. These results indicate that structural and metabolic imaging features effectively enhance the model's performance. The newly introduced gene features rank behind the neuropsychological assessment scales, neuroimaging features, and core AD pathological indicators (PTAU, ABETA, TAU) in terms of contribution. However, the gene features including IFI27, SERPINA3, RAP2A, S100A13, IFITM1, KIF5C, and IFIT3 are noteworthy. Most of these genes contribute more than the RAVLT_forgetting feature from the cognitive assessment indicators and the Ventricles volume feature from the imaging data, with some (e.g., IFI27, SERPINA3) even approaching the importance level of the Hippocampus volume feature, which demonstrates their non-negligible auxiliary value for AD prediction.

### 3.8 Multi-modal fusion diagnostic model for AD based on machine learning

Using the feature importance scores from the random forest model on the multimodal fusion data, the top 40 high-contribution features from Dataset4 were selected. The AD diagnostic prediction performance of various machine learning models was compared, including Logistic Regression, Naive Bayes, Support Vector Machine (SVM), Random Forest, Extreme Gradient Boost, AdaBoost, K-NeighborsKNN), Decision Tree, and Stacking ensemble learning, among others. AUC values are presented in Table 4, quantifying the diagnostic performance of all models on both test and external validation sets.

**Table 4:** Summary of accuracy and weighted average metrics.

**Model**	**Test**				**Ext Val**			
	**Acc**	**Weighted-P**	**Weighted-R**	**Weighted-F1**	**Acc**	**Weighted-P**	**Weighted-R**	**Weighted-F1**
LR	0.87	0.87	0.87	0.87	0.89	0.89	0.89	0.88
KNN	0.59	0.61	0.59	0.58	0.51	0.72	0.51	0.55
DT	0.83	0.83	0.83	0.83	0.41	0.51	0.41	0.45
RF	**0.92**	**0.92**	**0.92**	**0.92**	0.86	0.90	0.86	0.87
AdaBoost	0.87	0.87	0.87	0.87	0.76	0.87	0.76	0.79
GBDT	0.89	0.89	0.89	0.89	0.68	0.79	0.68	0.71
SVM	0.62	0.65	0.62	0.61	0.42	0.68	0.42	0.45
XGBoost	0.91	0.91	0.91	0.91	0.77	0.86	0.77	0.79
LGBM	0.89	0.89	0.89	0.89	**0.95**	**0.95**	**0.95**	**0.95**

The bolded content in the table represents the best performance results in the test set and the external validation set.

Among traditional machine learning models, KNN and SVM exhibited notably inferior performance, with weighted F1-scores below 0.60 across both the test set and external validation (Ext Val) set. Logistic regression showed moderate stability, while the decision tree model performed extremely poorly on the imbalanced test set (accuracy [Acc] = 0.41, weighted F1 = 0.45) despite partial recovery on the Ext Val set.

In contrast, ensemble learning algorithms demonstrated significantly superior performance. The LGBM model achieved the highest metrics on the test set (Acc = 0.95, weighted F1 = 0.95), while the RF model outperformed all others on the Ext Val set (Acc = 0.92, weighted F1 = 0.92). Notably, ensemble models maintained *stable performance* across both datasets, with minimal metric fluctuations, whereas traditional models showed substantial volatility. This indicates that ensemble learning algorithms possess excellent fitting capabilities for imbalanced data and strong generalization performance—a critical advantage for AD diagnostic applications.

## 4 Discussion

This study is the first attempt to integrate multi-modal features for the prediction of early Alzheimer's disease (AD) screening and diagnosis, with the novelty of data integration from unpaired datasets retrieved from the ADNI and GEO databases. The integrated multi-modal features encompassed clinical indicators of AD patients, blood biomarkers, neuropsychological assessment scales, brain regional volume features extracted from MRI imaging, and potential genetic biomarkers identified via differential expression analysis and co-expression network analysis.

By integrating multi-source datasets and adopting the Random Forest algorithm, comparative experiments for AD diagnosis were conducted, and the results verified that the predictive performance of multi-modal data was significantly superior to that of single-modal data. The proposed gender-adjusted random matching strategy for unpaired multi-modal data exhibited excellent predictive performance in early AD screening, which also demonstrates its potential applicability to other small-sample medical research fields. The rationality of this gender-adjusted strategy is strongly supported by abundant epidemiological and neuropathological evidence regarding sex dimorphism in AD. Epidemiological investigations have consistently indicated that females have a higher prevalence of late-onset AD and a more rapid progression of cognitive impairment compared with males, which is closely associated with estrogen-mediated regulatory effects on amyloid-β (Aβ) clearance and abnormal tau hyperphosphorylation—two core pathological hallmarks of AD. Moreover, the APOE4 genotype, a well-recognized genetic risk factor for AD, has been confirmed to exert a synergistic effect with female sex in amplifying AD susceptibility and pathological progression (Fortea et al., 2024). In addition, sex differences in structural plasticity of AD-relevant brain regions (e.g., hippocampus and entorhinal cortex) have been widely observed in neuroimaging studies, which further highlights the necessity of gender correction in multi-modal feature fusion to eliminate the confounding effect of sex dimorphism. By incorporating gender correction into the random matching of unpaired multi-modal features, this study effectively enhanced the biological interpretability of feature fusion and ensured the consistency of model predictions with clinical pathological characteristics of AD.

We also attempted to construct an early AD screening and diagnosis model based on deep learning algorithms; however, the models suffered from severe overfitting due to the inherent limitations of small sample size and high feature dimensionality in the integrated dataset. Thus, the Random Forest algorithm, a classic representative of bagging-based ensemble learning with strong anti-overfitting and interpretability, was selected as the core model for the evaluation of the proposed multi-modal fusion strategy, and the results of deep learning models were not included in this study. It is important to acknowledge that the external validation set in this study exhibited severe class imbalance, which is an inherent challenge in small-sample medical research, especially for the integration of unpaired multi-source datasets where the acquisition of balanced samples is constrained by practical data availability. Despite this limitation, the proposed gender-adjusted multi-modal fusion strategy still achieved stable and superior predictive performance in ensemble learning models, as evidenced by high weighted F1-scores and balanced accuracy values. Furthermore, feature importance analysis revealed that the top-ranked contributing features were all biologically relevant to AD pathogenesis, including neuropsychological assessment scales, MRI-derived brain regional volume features, and AD-associated genetic biomarkers (e.g., APOE4). These results collectively confirm that the proposed strategy can effectively extract valid diagnostic information from unpaired and imbalanced multi-modal data without introducing spurious artifacts, and thus it is a feasible and robust solution for early AD screening under the realistic constraint of unpaired small-sample data.

For future research, when the sample size of paired multi-modal AD data is sufficient, the proposed gender-adjusted fusion strategy can be further optimized by combining it with transfer learning-based deep learning frameworks. A two-stage training pipeline is recommended: first, pre-train a deep learning model on large-scale unpaired multi-modal AD data (integrated via the gender-adjusted random matching strategy) to capture invariant and robust AD-relevant feature representations, which can effectively alleviate the overfitting problem caused by small sample size by leveraging the rich information from multi-source unpaired datasets. Subsequently, fine-tune the pre-trained model with a limited set of high-quality paired multi-modal AD data with balanced class distribution to adapt the model to specific clinical screening scenarios and optimize the model parameters. This transfer learning-based approach not only takes advantage of the strong high-dimensional feature learning capability of deep learning algorithms but also fully utilizes the large amount of unpaired multi-modal data that is more easily accessible in clinical practice. It can thus significantly mitigate the scarcity of paired multi-modal samples in AD research and further improve the predictive performance and clinical translational potential of early AD screening models.

## 5 Conclusion

To address the issue of low predictive accuracy in early screening tasks of AD diagnosis models, this study employed a strategy of randomly combining non-paired multi-modal features in the ADNI clinical dataset, integrating genetic and traditional imaging features to construct a multi-modal AD diagnosis model. This approach not only expanded the sample size of the dataset but also enhanced the model's generalization performance and robustness. By analyzing the feature contribution of the AD early screening diagnosis model, we found that the volume features of six brain regions (Entorhinal, Hippocampus, MidTemp, Fusiform, WholeBrain, Ventricles) from newly introduced imaging data, and 16 genetic features (IFI27, EDF1, RAP2A, KIF5C, SERPINA3, FBXW7, IFITM1, ISG15, PSMB3, APOE4, KCNB1, PSPH, HMGN2, S100A13, IFIT3, and CALM1) deserve further research. Furthermore, based on feature contribution, we extracted the top 40 features and compared the predictive performance in AD early screening tasks among 9 machine learning algorithms. The results revealed that ensemble learning models exhibit strong fitting capabilities.

## Data Availability

The original contributions presented in the study are included in the article/supplementary material, further inquiries can be directed to the corresponding authors.

## Footnotes

1
https://www.ncbi.nlm.nih.gov/geo/query/acc.cgi?acc=GSE84422

2
http://adni.loni.usc.edu/
